# Multiple factors influence claw characteristics in oribatid mites (Acari)

**DOI:** 10.1038/s41598-024-58214-4

**Published:** 2024-04-02

**Authors:** Michaela Kerschbaumer, Tobias Pfingstl

**Affiliations:** Institute of Biology, University of Graz, Universitaetsplatz 2, 8010 Graz, Austria

**Keywords:** Ecology, Evolution, Zoology

## Abstract

Claws, as nature's multifaceted instruments, play fundamental roles across the animal kingdom, aiding in prey capture and enabling movement across diverse terrains. Claw features often reflect the ecologies of the respective taxa and thus can provide important insights into the different lifestyles. This study explores the claw morphology of monodactylous oribatid mites through geometric morphometrics, analyzing 559 specimens from 49 species across various ecosystems. The research identifies distinct claw characteristics associated with specific habitats, revealing a significant correlation between claw morphology and the mites' environmental adaptations. Littoral mites exhibit notably larger claws compared to terrestrial counterparts, with aquatic and semiaquatic species presenting intermediate traits. The analysis shows an inverse relationship between claw curvature and sharpness, differing from patterns observed in larger animals. A trend of increasing claw bluntness with body size in terrestrial mites echoes biomechanical constraints seen in larger species. The study also observes consistent claw shapes within oribatid superfamilies, suggesting a potential, albeit muted, phylogenetic influence alongside environmental factors. These findings reveal how ecological, evolutionary, and functional aspects influence claw morphology in oribatid mites, enhancing our knowledge of arthropod biology and potentially inspiring biomimetic advances in material science and engineering.

## Introduction

Claws, versatile biological tools, are found across a broad spectrum of the animal kingdom, from the minuscule arthropods to the large mammals. These biological appendages serve a multitude of functions, including but not limited to, digging, climbing, and capturing prey^1^. The morphology of these claws, particularly their curvature and size, often provides a window into the ecological niches and lifestyles of the species^2^. For instance, studies on avian and reptilian species have shown that tree-dwelling species tend to have more curved and taller claws compared to their ground-dwelling counterparts^3–5^. Such morphological insights have even been instrumental in interpreting the ecological behaviors of extinct reptilian species from their fossilized remains^6,7^. Given that claws serve as the primary gripping mechanism in vertebrates^5^, a considerable number of research has been conducted on their comparative anatomy and morphology. This research is especially prevalent in reptiles^5,8–12^ and birds^6,13^. In the invertebrate world, which is packed with species possessing claws, claw morphologies are extremely diverse and the correlation between attachment and morphological structure is more complex. For example, most insects and some mites possess adhesive pads or other structures, in addition to the claws, to maintain grip on a variety of surfaces (e.g.^14,15^. The pads are mainly used to cling to smooth surfaces while the claws allow attachment to rougher ground^16^. Büscher and Gorb's seminal work on stick insects^17^, elucidated this intricate interplay between claws and adhesive pads, revealing how these structures are fine-tuned to the substrate surfaces these insects encounter. Other insects are specialized to adhere only to certain substrates and therefore show very specific adaptations of their attachment devices. Parasitic dipteran flies, for example, must stay attached to the body of the host and consequently have evolved dentate or comb-like claws, and dichotomously shaped setae on their pads, which allow extreme strong attachment, but only to the host’s body^18,19^.

The morphology of claws and additional attachment devices may also change during development in certain arthropods. Scorpions, whip-scorpions, and whip spiders, show maternal care and their prenymphs possess specific adhesive pads to stay on their mother’s back, but these organs are reduced and replaced by claws in the following free-living nymphal and adult stages^20^. In the development of the giant stick insect *Eurycantha calcarata*, a nuanced adaptation in attachment mechanisms aligns with ontogenetic behavioral shifts. While nymphs display developed adhesive pads for arboreal navigation, reflecting their leaf-dwelling phase, adults exhibit more pronounced claws, suited for terrestrial mobility around roosting areas. This transition suggests a functional evolution in locomotion strategies, as detailed by Gottardo et al.^21^ and Boisseau et al.^22^. The integration of these findings points to a complex interplay between morphological and behavioral adaptations within their ecological niche.Due to this diversity, complexity and possible ontogenetic changes of attachment devices in arthropods, it is difficult to perform studies that elucidate how these structures interact with the environment and how ecology and evolution have shaped them. In this respect, mites, the tiniest members of arthropods, present a unique opportunity for ecomorphological studies. Their diminutive size means they interact with a microcosm of ecological niches, each with its distinct microstructural landscape. Especially oribatid mites, also known as moss mites, inhabit all soil-type habitats from tropical rainforest to polar deserts^23^. Moreover, the vast majority of Oribatida only possesses claws and no additional adhesive structures^15^, which allows to focus on the interaction of the claws with the environment. On that note, the first geometric morphometric study on claw shapes of intertidal oribatid mites^24^ revealed a clear correlation between claw shape and inhabited substrate. Those dwelling on hard surfaces like rocky coastal environments have distinctly higher and more curved claws compared to those found on softer terrains like mangrove roots and litter. An intermediate claw morphology is present in species that have a broader substrate range. The tidal zones, these mites inhabit, subject them to the relentless forces of tidal water movements, which in turn have driven the evolution of their claw morphology to optimize movement and attachment. The strong evolutionary pressure in this extreme environment even has led to the loss of a phylogenetic signal in the claws of intertidal oribatid mites^25^. However, recent initial studies on claws of a few typical terrestrial oribatid mite species^26,27^ indicated that habitat specificity and lifestyle also have an impact on claw morphology in the terrestrial environment, but this correlation is apparently weaker than in intertidal oribatid mites which are subject to strong external forces. Moreover, a phylogenetic signal in the shape of claws could be quantified at least in one group of these terrestrial oribatid mites^27^. These recent studies on terrestrial oribatid mites provided interesting first insights but were by far not comprehensive enough to unravel the factors shaping claw morphologies in the terrestrial environment.

The present study aims to delve deeper into the world of terrestrial oribatid mites, by expanding the scope to encompass a wider range of species and ecological niches. By using a comprehensive geometric morphometric examination of claw traits across various monodactylous (possess only a single tarsal claw on each leg) oribatid families from different ecosystems, we try to better understand the intricate correlation between claw characteristics, ecosystem, and relationship.

## Methods

### Specimen source

We examined claw characteristics in a total of 559 specimens representing 49 distinct species, spanning across 16 oribatid superfamilies (Table 1). The specimens were either collected by the authors between 2020 and 2023 or sourced from the oribatid mite collection housed at the Institute of Biology, University of Graz (IBUG), Austria.

**Table 1:** Examined oribatid mite species, with body length, claw shape, claw curvature and sharpness.

Superfamily	Species	n	Min–max bl (µm)	Claw lateral	Gamma	Tip.gamma
Hypochthonioidea	*Mesoplophora pulchra*	9	263–283		116	46
Phthiracaroidea	*Steganacarus applicatus*	8	826–1020		105	72
Crotonioidea	*Platynothrus peltifer*	23	785–991		81	64
	*Malaconothrus monodactylus*	6	355–406		87	56
	*Hermannia convexa*	7	1208–1451		94	65
	*Hermannia gibba*	21	584–915		89	67
	*Nanhermannia coronata*	11	516–570		88	49
Ameroidea	*Damaeolus asperatus*	14	223–262		99	54
	*Fosseremaeus laciniatus*	12	221–260		103	49
Damaeoidea	*Porobelba spinosa*	10	409–482		102	60
Licneremaeoidea	*Lamellovertex caelatus*	14	338–471		95	53
Oppioidea	*Oppiella nova*	6	248–274		94	59
Oripodoidea	*Liebstadia similis*	6	490–562		94	74
Tectocepheoidea	*Tectocepheus v. sarekensis*	15	322–373		91	58
Cepheoidea	*Cepheus cepheiformis*	9	642–802		100	63
Caleremaeoidea	*Caleremaeus alpinus*	24	340–411		86	71
	*Caleremaeus lignophilus*	29	294–373		90	64
	*Caleremaeus mentobellus*	22	322–388		91	55
Carabodoidea	*Carabodes areolatus*	46	432–611		89	61
	*Carabodes coriaceus*	15	698–823		95	63
	*Carabodes reticulatus*	7	642–795		98	64
	*Carabodes rugosior*	26	490–711		84	62
	*Odontocepheus elongatus*	16	514–731		90	51
	*Dolicheremaeus dorni*	7	544–625		104	52
Ceratozetoidea	*Mycobates carli*	20	356–425		98	53
	*Mycobates parmeliae*	14	458–922		88	70
Limnozetoidea	*Hydrozetes lemnae*	25	440–560		88	69
Ameronothroidea	*Alismobates galapagoensis*	9	331–372		99	63
	*Alismobates inexpectatus*	25	346–399		88	80
	*Fortuynia hawaiiensis*	8	387–420		83	79
	*Fortuynia rotunda*	13	607–662		87	69
	*Litoribates bonairensis*	18	326–374		97	60
	*Litoribates caelestis*	10	295–325		92	68
	*Carinozetes mangrovi*	7	340–363		102	61
	*Thalassozetes balboa*	10	278–320		89	82
	*Thalassozetes grenadensis*	8	261–292		89	86
	*Thasecazetes falcidactylus*	5	370–417		107	66
	*Tegeocranellus knysnaensis*	13	266–294		106	61
	*Tegeocranellus sacchareus*	11	328–350		100	71

Species were classified into four distinct groups according to their presence in different ecosystems: terrestrial, littoral, aquatic, and semiaquatic. This classification is based on widely accepted literature^28–30^. Taxonomic classification follows that of Norton and Behan-Pelletier^23^ and Behan-Pelletier and Lindo^31^.

### Geometric morphometrics and measurements on claw

**Figure 1:**
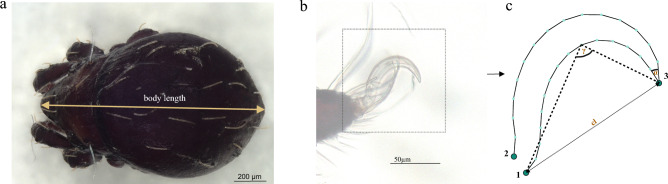
**(a)** Habitus of *Hermannia convexa* with body length measurement scheme and its (**b)** claw on the first leg **c** Landmarkset and linear as well as angular measurements on claw.

**Figure 2:**
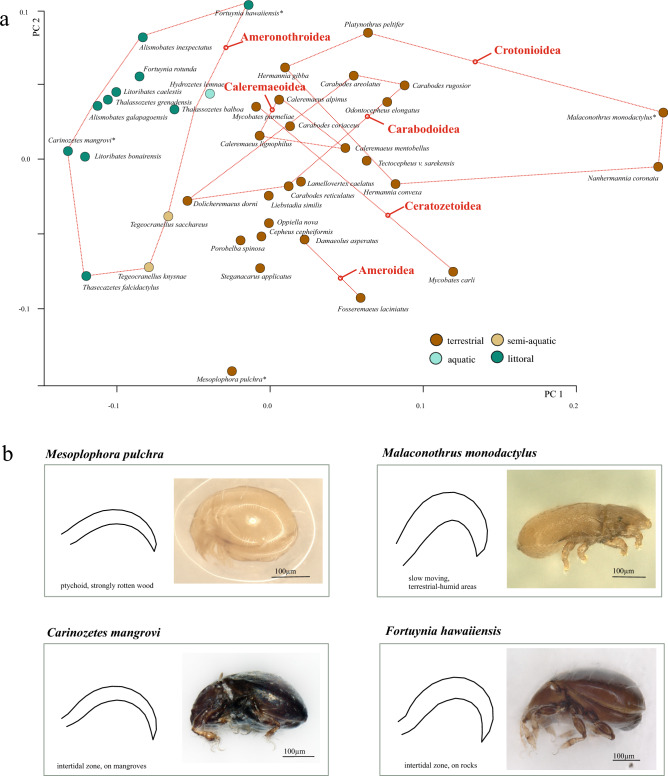
**(a)** PCA of oribatid species claw mean shapes with focus on ecosystem and superfamily mapped onto the first two principal components (PC1 and PC2). Species are connected by dotted lines to denote their grouping into specific families. Color-coding of data points indicates their ecosystem (**b)** Species showing outstanding claw shapes within the dataset, species are marked with * in PCA scatterplot.

In earlier investigations, we developed innovative methodologies for quantifying claw characteristics in mites using geometric morphometrics (GM). For a comprehensive description of our specimen preparation, photography, landmark selection, and coordinate data processing, please refer to Pfingstl et al.^24^ and Kerschbaumer et al.^27^. In addition to claw shape, claw length, and claw curvature (γ), we incorporated the morphological parameter, claw sharpness, into our analysis. This was achieved by measuring the claw tip angle (α) of the mite claws (Fig. 1). All our analyses were executed using R, version 4.2.2^32^. Landmark digitization was carried out using TpsDig2, Version 2.31^33^, and raw coordinates were subsequently imported into R and we did the Generalized Procrustes Analysis (GPA) on our landmarks and semi-landmarks (using function gpagen). All configurations were translated, normalized and rotated to minimize the overall sum of the squared distances between the corresponding landmarks and semilandmarks (Fig. 2). To remove the effects of scale, GPA also computes a unit centroid size as the square root of the summed squared distances from all landmarks and semilandmarks to their centroid^34^. The following R-packages were employed for subsequent analyses: "ggplot2," "geomorph," "Morpho," and "shapes"^35–38^. Mean shape of aligned specimens was calculated for each species using the "mshape ()" function within the geomorph package. Subsequently, a Principal Component Analysis (PCA) was conducted on the mean claw shapes of various species. From this analysis, we got the four most distinct claw shapes and compared them in terms of ecological aspects. To explore the connection between structural habitat preferences and claw morphology, we categorized species into four distinct ecosystems (terrestrial, semi-aquatic, aquatic, and littoral). To quantify differences in claw characteristics across ecosystems and superfamilies, we employed Procrustes ANOVA^39^ for shape data and conducted one-way ANOVA tests along with Tukey's Honestly Significant Difference (HSD) post-hoc tests for angular and linear measurements of claws. In our analysis, we employed the 'geom_smooth' function with the 'method = lm' parameter in R. This method utilizes linear regression to fit a trendline to our data, providing a visual representation of the linear relationship between the variables of interest. This approach allowed us to assess the linear association between claw curvature and claw sharpness and body size and claw sharpness, respectively.

## Results

**Figure 3:**
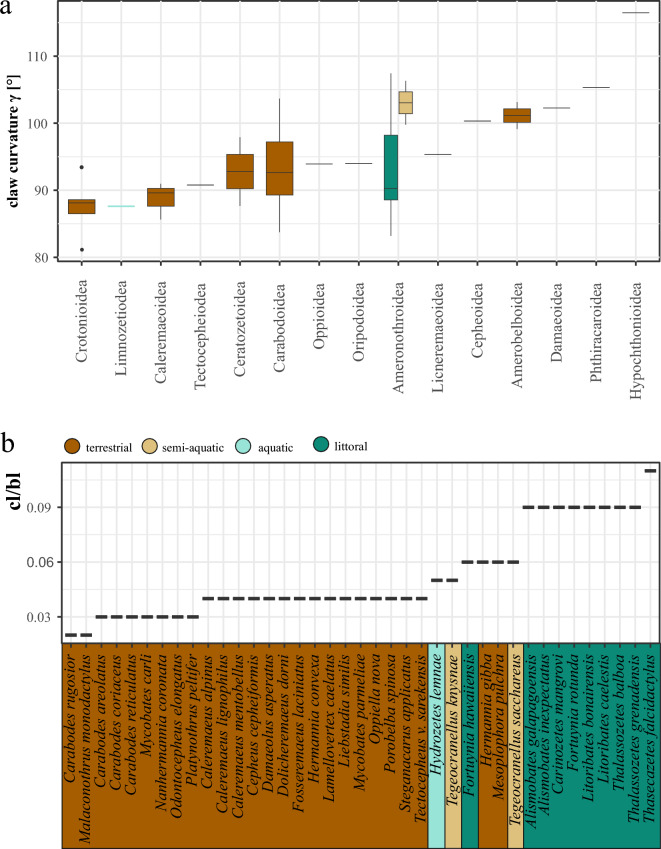
**(a)** Box plot illustrating the distribution of claw curvature (measured in degrees) across various oribatid mite superfamilies. (**b)** Bar chart showing the ratio of claw length to body length (cl/bl) for individual oribatid mite species. Species are color-coded based on their ecosystem.

**Figure 4:**
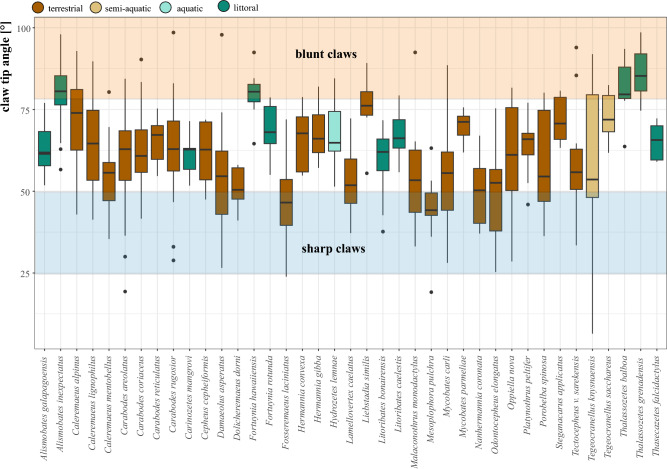
Sharpness of the claw. Boxplot of measurements of the claw tip angle (measured in degrees) across various oribatid mite species. Boxes are color-coded based on species ecosystem.

**Figure 5:**
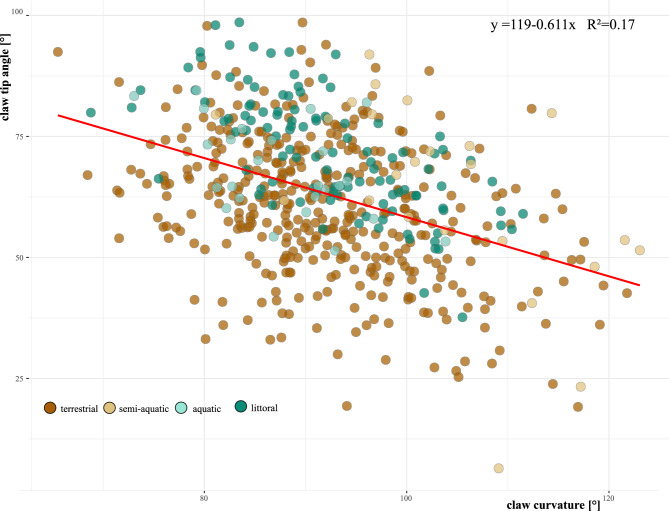
Correlation between the claw tip angle (in degrees) and the claw curvature (in degrees) for various oribatid mite species. Each dot indicates an individual mite sample, with their ecosystem represented by distinct colors.

Our examination of claw characteristics across the 49 studied species of oribatid mites unveiled a remarkable diversity in claw shapes (Table 1). Claw curvatures range from 81° (strongly curved) in *Platynothrus peltifer* to 116° (very flat) in *Mesoplophora pulchra.* The latter also shows the sharpest claw tip with an angle of 46° and the bluntest claw is shown by the littoral *Thalassozetes grenadensis* with a tip angle of 86°. We can observe highly robust claws in species such as *Malaconothrus monodactylus*, contrasting with the very fine claws seen for example in *Oppiella nova* (Table 1). The classification of the examined species based on the ecosystem has yielded that oribatid mites residing within the same ecosystem tend to exhibit more similar claw forms, with littoral mites standing out as readily distinguishable from their terrestrial counterparts. Procrustes ANOVA revealed a statistically significant difference in claw shape among ecosystems (Table 2a), with relative claw size cl/bl showing the statistically most significant difference. This difference in cl/bl according to ecosystem is confirmed by Tukey post-hoc test revealing significant pairwise differences between littoral species and species of the other three ecosystems. Species of littoral mites possess disproportionately larger claws relative to their body size compared to terrestrial species, and aquatic and semi-aquatic claws are in between, concerning the measurement cl/bl (Fig. 3b). The measurements claw curvature and claw tip angle, on the other hand, show no significant differences between littoral species and species of the other three ecosystems (Table 2b, Fig. 3a). A closer examination of claw sharpness unveils an interesting pattern: littoral species that inhabit hard substrates (*Thalassozetes balboa, T. grenadensis, Alismobates inexpectatus* and *Fortuynia hawaiiensis*) tend to have the bluntest claws (claw tip angle > 80°). The sharpest claws (claw tip angle < 50°) are found in terrestrial species (*M. pulchra*, *Nanhermannia coronata*, *Fosseremus laciniatus*) (Table 1, Fig. 4). Most species (69%) have claws with a tip diameter between 50° and 75°. Additionally, we observed an inverse relationship between claw curvature and sharpness in all species independent of their ecosystem affiliation, with stronger curved claws showing blunter tips and flatter claws displaying sharper tips, demonstrated by a linear regression in Fig. 5 (y = 119 − 0.611; R^2^ = 0.17). In terrestrial species only, we detected an increasing claw tip angle with growing body size. The linear regression showed a correlation between the sharpness and size of mites, with a regression coefficient of 0.21 and a p-value less than 0.05 (Fig. S1).

**Table 2:** Results of Procrustes ANOVA on claw shape and Tukey multiple comparisons of the measurements cl/bl, claw curvature and claw tip angle among ecosystems. *p values significant at the 5% level.

Claw shape	Df	SS	MS	Rsq	F	Z	p	
Ecosystem	3	0.17403	0.05801	0.37002	6.8526	3.5283	0.001*	
Residuals	35	0.29629	0.008465	0.62998				
Total	38	0.47032						

clbl	Diff	*p*	Claw curvature	Diff	*p*	Claw tip	Diff	*p*
Littoral-aquatic	0.039	0.0035*	Littoral-aquatic	5.704	0.8951	Littoral-aquatic	− 0.003	0.9989
Semiaquatic-aquatic	0.005	0.9761	Semiaquatic-aquatic	15.437	0.3756	Semiaquatic-aquatic	0.027	0.5918
Terrestric-aquatic	− 0.013	0.5974	Terrestric-aquatic	6.622	0.8349	Terrestric-aquatic	0.006	0.9833
Semiaquatic-littoral	− 0.34	0.0005*	Semiaquatic-littoral	9.732	0.3784	Semiaquatic-littoral	0.029	0.1447
Terrestric-littoral	− 0.052	0.0000*	Terrestric-littoral	0.918	0.9886	Terrestric-littoral	0.009	0.5045
Terrestric-littoral	− 0.018	0.0902	Terrestric-littoral	− 8.814	0.4178	Terrestric-littoral	− 0.02	0.3898

Within the superfamilies we studied, there is a notable consistency in claw shape among different species. For example, in the Ameroidea, *Damaeolus asperatus* and *F. laciniatus* have claws that are flat and comparatively thin. In contrast, most species within the Crotonioidea superfamily possess claws that are thick, powerful, and distinctly curved (Table 1). Besides this trend, there are also outliers in other families. In the case of the superfamily Carabodoidea, where members basically show strongly curved (84–95°) and robust (higher from ventral to dorsal edge) claws, *Dolicheremaeus dorni* and *Carabodes reticulatus* stand out distinctly with less curvature (98–104°) and lower claw heights (Table 1). Among terrestrial species, *M. pulchra* and *M. monodactylus* show the most distinctive claw shapes but in opposite directions, *M. pulchra* possesses a very flat and delicate claw, while *M. monodactylus* exhibits a strongly curved and extremely robust claw (Fig. 2b). In the littoral environment, it is *Carinozetes mangrovi* showing a very flat and rather delicate claw while *F. hawaiiensis* possesses the strongest curvature and height (Fig. 2b). *M. pulchra* occurs in dead decaying wood, *M. monodactylus* dwells in wet meadows, bogs and forest soils, *C. mangrovi* can be found in mangrove forests and *F. hawaiiensis* inhabits rocky harsh coasts. So, these species show all very different ecologies which is reflected in their claw shapes.

## Discussion

### Ecosystem and claw characteristics

In our study, we classified the species into four distinct groups based on their presence in different ecosystems: terrestrial, littoral, aquatic, and semiaquatic. It is well-established that the majority of Oribatida are terrestrial in nature, displaying a preference for moist terrestrial habitats, while only a limited number of species have adapted to aquatic environments^29,40^. The present results clearly demonstrate that species sharing the same ecosystem tend to possess certain similar claw characteristics, which supports the notion that environmental pressures sculpt morphological traits. Littoral oribatid mites possess claws nearly twice the size compared to their terrestrial counterparts. This pronounced difference between littoral and terrestrial mites, with aquatic and semiaquatic species falling in between, resonates with previous findings on claw shape and microhabitat relationships in intertidal mites^24,25,41^. The extreme conditions of the intertidal zone with the moving tidal water, faced by littoral species, have largely influenced this unique adaptation of having considerably larger claws^24^. Larger claws might provide more surface area for attachment, countering the powerful forces of tidal water movements. From that perspective, it can also be explained why aquatic and semiaquatic species show intermediate claw lengths. They are temporarily or permanently flooded in limnic environments which are not subject to strong water movement, except for rivers and other running waters. Keeping attached under flooded conditions is, due to buoyancy and water resistance, probably more difficult than to stay attached in atmospheric air, but still easier than to fight against tidal surf and wave action. It seems to be a general rule that attachment in environments, which are strongly influenced by water and water movement, requires relatively longer claws, at least in smaller arthropods.

The characters claw curvature and sharpness, on the other hand, are apparently not strongly influenced by the factor water as they do not significantly differ among the members of different ecosystems.

### Claw curvature

Earlier studies on claw shapes of intertidal oribatid mites^24,25^ demonstrated that claw curvature is strongly affected by the type of substrate the mites walk on. Strongly curved claws are found in rocky habitats while less curved claws are typical for environments with softer surfaces, like mangrove roots and leaves^24^. Given the wide variation in claw curvature observed among terrestrial oribatid mites, it is plausible to assume that this diversity correlates with the variety of substrates found in terrestrial environments. Until now, we have not conducted a direct study of the substrate characteristics. However, we aim to explore this aspect more thoroughly in future studies to make clearer statements about the correlation between claw morphology and substrate. Anyway, an initial study on the claws of terrestrial *Caleremaeus* and *Carabodes* could not infer such a direct correlation but concluded that strong habitat specificity may have a considerable impact on claw shape, whereas the nature of this impact remained unsolved^27^. The present study provides further interesting insights but is also unable to infer a clear correlation between substrate and claw shape as shown in intertidal oribatid mites. Considering the terrestrial species that show claw curvatures higher than 100°, meaning they have the least curved claws, they all occur in habitats with softer substrates, for example *F. laciniatus* is often found in soils of deciduous forests, *Cepheus cepheiformis* in forest soils and peaty ground^28^ and *D. dorni* occurs in decaying leaves, dead wood and tree-associated mushrooms^42^. This would be well in accordance with the results from littoral environments^24^, i.e. less curvature is linked with softer substrates. But if we look at the species with claw curvatures smaller than 90°, which means their claws are strongly curved, only *Caleremaeus alpinus* is known to predominantly occur in mosses on rocks and thus on hard substrates^27^. All the other species dwell in habitats with softer substrates, e.g. *Platynothrus peltifer* is often found in bog soils and wet forest soils, *Hermannia gibba* mainly lives in forest floors and bogs, and *Carabodes rugosior* often occurs in leaf litter and on tree stumps etc. Moreover, the purely rock-dwelling *Lamellovertex caelatus* shows an intermediate curvature with 95°, consequently, the correlation between curvature and substrate properties in terrestrial oribatid mites is not exactly the same as found in intertidal oribatid mites and other influencing factors should be considered.

In terrestrial mites, claw curvatures exceeding angles of 100°, i.e. flat claws, almost exclusively occur in superfamilies that exhibit a somehow deviating body or leg morphology (see Fig. 3a). The Cepheoidea possess large round, almost spherical bodies with relatively short legs, the Ameroidea and Damaeoidea are all characterized by remarkably long and thin legs, and the Phthiracaroidea and Hypochthonioidea (which are herein only represented by *M. pulchra and Steganacarus applicatus*) are ptychoid mites or so-called ‘box mites’ mites, which means they are able to retract their legs into the opisthosoma and close like a seed as a defensive adaptation^31^. All the other herein investigated terrestrial oribatid mites, with curvatures less than 100°, are more or less, similar in body form and body leg length ratio. Consequently, deviations from this ‘common’ body form and leg morphology scheme may have an influence on claw curvature, as well. Detailed studies, including leg lengths, size ratios and body form, are clearly needed to elucidate this possible correlation and the mechanics behind it.

Another factor that may shape claw curvature is the distinct locomotor mode the mites use. Studies in lizards and turtles^4,5,8^ have shown that climbing species generally have more curved claws than ground-dwelling species. Another study defined eight functional categories of claws, e.g. amplectorial (grasping), cursorial (running), scalporial (scratch digging) etc. and demonstrated that claw function can be inferred from claw morphology with high confidence in birds, reptiles and mammals^43^. For intertidal oribatid mites, attachment may be the most important function of claws, but for terrestrial mites other functions may also be of essential relevance. Arboreal mite species, for example, must be excellent climbers and it is assumable that some other species may even use their claws for digging. However, apart from a single study^44^, direct observations on claw function are yet lacking in oribatid mites and therefore it is not possible to establish functional categories and to relate them to specific claw curvatures, shapes respectively, as it was done by Thomson & Motani^43^. Nevertheless, it is assumable that some mite species often migrate vertically and thus tend to climb frequently which probably results in stronger curved claws, while others prefer to move in a horizontal direction and are thus simply walking on ‘flat ground’, requiring relatively flat claws. If claw curvature is related to a ‘climbing’ or a ‘walking’ lifestyle needs to be investigated in further studies, which should also include observations of living specimens.

### Claw sharpness

In our study, the measure of claw sharpness is derived from the claw tip angle, a methodology echoing that of Turnball et al.^45^. This approach contrasts with previous investigations on larger arthropods and vertebrates, where sharpness is frequently quantified using the claw tip radius^46,47^. The lateral 2D view we employ restricts us from determining the claw tip radius, and the minuscule size of mites renders the acquisition of a 3D claw representation currently unattainable. Despite these methodological differences, comparisons and discussions on the functional implications of claw sharpness across diverse taxa remain pertinent.

From the broader literature, the functional significance of claw sharpness is evident. Optimal sharpness enhances the likelihood of claws interlocking with small surface asperities, a crucial factor for effective attachment^48^. However, the biomechanical trade-offs are clear: while sharper claws confer superior interlocking capabilities, their structural delicacy makes them vulnerable to fracture and wear. This balance between sharpness for efficient attachment and thickness for durability is underscored by the study of Pattrick et al.^46^, where insects with sharper claws demonstrated superior surface attachment but were at heightened risk of breaking their claws.

The present results indicate that littoral oribatid mites generally tend to have blunter claws and considering the mechanical stress due to strong exposure in the littoral environment, it becomes evident that sharper claws entail too great a risk of breaking in this habitat. The bluntest claw tips are shown by rock dwelling species, like *A. inexpectatus*, *F. hawaiiensis*, *T. balboa* and *T. grenadensis*, their ‘blunter’ tips apparently reduce the risk of fracture and still provide the ability to grip onto the rough and coarse texture of the rocky shore habitat. Species mainly associated with mangrove habitats, like *Carinozetes mangrovi*, *Fortuynia rotunda*, *Litoribates bonairensis* and *Thasecazetes falcidactylus*, on the other hand, have sharper claws, which might be a consequence of being less exposed to strong wave action and having to cope with smoother substrates like mangrove leaves.

In insects and cursorial (running/hopping) vertebrates, it was shown that the relative bluntness of the claws increases with increasing body mass^43,46^, potentially compromising attachment performance. Although this trend is not present in littoral oribatid mites, it is mirrored in their terrestrial counterparts.

As terrestrial mites increase in size, their claws become blunter, as evidenced by the increasing claw tip angle (see Fig. S1). Claws of larger insects are predicted to experience increasing stress because weight increases faster than claw tip area, which can lead to breakage or wear^46^. In vertebrates, larger animals also tend to have more blunted claws, to provide a broader support for their increased mass^43^. The same principles seem to apply to the tiny mites as well, larger body sizes result in relatively more weight carried by each claw, which in turn heightens the risk of fracture. The fundamental challenges of achieving effective grip while mitigating risks of wear and fracture thus remain universal.

The present results further indicate that claw sharpness in oribatid mites is also correlated with claw curvature, lesser curved claws are basically equipped with sharper claw tips. This is in contrast to studies on vertebrates, where sharper tips are a feature of strongly curved claws of arboreal species and blunter tips are typical for long straight claws of terrestrial (cursorial) species^4,5,8,49^. Why the mites show the opposite correlation is yet unclear and needs further investigation. Considering the principle of scaling, as organisms grow in size, their weight increases faster than their strength due to weight being linked to volume (cubically scaling) and strength to the cross-sectional area (quadratically scaling). Hence, smaller arthropods like oribatid mites experience less impact from their weight on the form and function of their claws compared to larger insects. This scaling principle suggests that the variation in claw sharpness among arthropods of different sizes is a result of biomechanical constraints, underscoring the significance of physical principles in the morphological evolution and diversity of arthropods.

### Extreme terrestrial claw shapes

*M. pulchra,* features exceptionally sharp claws, with a claw tip angle of approximately 46°. In contrast, its claws are the least curved, with an angle of 116°. The Mesoplophoridae belong to the taxonomic group of Enarthronota within the 'Lower Oribatida'. This family of oribatid mites is characterized by the ability to retract their legs and podosoma into the hysterosoma, a process known as ptychoidy, with the dorsal part of the podosoma, referred to as the aspis, serving as a cover^23^. When the legs and associated structures are retracted, the mites form a perfectly armored ball or sphere showing no weak points for possible predatory attacks. This also means, they need to quickly cease attachment to the ground during this defensive behavior. The ability to quickly detach and the reduced need for staying permanently attached apparently resulted in the hardly curved thin claws. The extremely sharp tip of the claws may still provide effective attachment during motion and thus compensate for the reduced claw curvature and height. In Austria, *M. pulchra* has been primarily found in substrates consisting of heavily decomposed wood, rich in coprogenous humus and fecal pellets from larger wood-feeding arthropods^50^. It appears that *M. pulchra* is a stenotopic species regarding substrate type and the used substrates are basically softer materials, which could also explain the lower curvature of their claws. If *M. pulchra* really exclusively favors decaying wood within specific stages of decomposition, questions arise regarding how these animals move to new suitable habitats. The very sharp and flattened claws may have a limited role in this process. On the one hand, they are not well-suited for grasping during possible phoretic dispersion, and on the other hand, their sharp and delicate structure renders them unsuitable for prolonged migration. However, a very similar claw shape is shown in *Steganacarus applicatus*, a species often occurring in forest soils. This species also shows a ptychoid body, but here the defensive mechanism has evolved independently from the Mesoplophoridae and represents a convergent adaptation^23^. This indicates that this specific body form apparently requires a very specific claw shape, i.e. weak curvature, low height and sharp tip.

The claw morphology of *Malaconothrus monodactylus* is particularly intriguing, as it shows relatively short claws with a strong curvature, considerable height and sharp tips. This species favors wet meadows, bogs, forests, and the peripheries of forest lakes^28^, exhibits a deliberate pace, moving considerably slower than many of its peers. Its claw design closely mirrors that of its relatives, *Nanhermannia coronata* and *Platynothrus peltifer*, both also showing preferences for wetter habitats^28^. They all belong to the superfamily Crotonioidea, which has the most prominent claw curvature in our dataset. The claw characteristics shown by these mites are typical for climbing species in vertebrates^5,8^, therefore, it could also be the case that these mites tend to show frequent vertical movement in their microhabitat, but observations in this respect are lacking. Strong claw curvatures are also known to be shown in digging tetrapod vertebrates^9,43^ and it could also be possible that these crotonioid mite species dig deeper into the ground to avoid desiccation when their wet habitats become dry, similar to mite taxa from ephemeral rock pools^51^. But again, observations of such a behavior are lacking so far.

As mentioned in the earlier discussion, environments subject to water apparently require relatively longer claws to provide firm grip. These crotonioid species often occurring in wet habitats show the shortest claws in our dataset, which raises the question how they ensure firm attachment while being flooded. In contrast to the littoral and most semiaquatic species, these Crotonioidea possess thickened and barbed distal tarsal setae which are supposed to support the claws in attaching to the substrate during submergence in water^15^ and this could explain the shortness of their claws.

### Phylogeny and claws

Aside from environmental variables, taxonomic relationships may have a substantial impact on morphological form. Strong phylogenetic signals, which means related species tend to relate each other morphologically more than any randomly chosen taxon^52^, were, for example, reported in the wing shape of certain dipterans^53,54^ or the anchor shape of parasitic monogeneans^55^. The claw shape of littoral oribatid mites, on the other hand, is completely lacking a phylogenetic signal, possibly due to the strong environmental pressure exerted on this morphological trait in this extreme habitat^25^. In the terrestrial oribatid mite genus *Carabodes*, which is included here, a phylogenetic signal in the claws could be quantified, but at the same time it was absent from another terrestrial mite genus, namely *Caleremaeus*^27^. Therefore, the phylogenetic influence on the structure of claws of terrestrial oribatid mites remained inconclusive. Although the present study does not include molecular genetic data, the results indicate that the influence of phylogeny may be present but it is rather weak. Claw shapes within the Crotonioidea are all basically similar and could indicate the presence of a phylogenetic signal, but nearly all herein investigated species show similar or largely overlapping ecologies. The Carabodoidea also exhibit similar claw traits and the above-mentioned study^27^ demonstrated that a phylogenetic signal is present in the genus *Carabodes*. However, *D. dorni*, which was not included in the latter study^27^, but is a member of this superfamily, strongly deviates in its claw characteristics. Moreover, the Hypochthonioidea and Phthiracaroidea, with *M. pulchra* and *S. applicatus*, show strikingly similar claw shapes, but are not closely related, suggesting that their convergently evolved ptychoid body form and not phylogenetic relatedness is responsible for their similar appearance. On a higher taxonomic level, claw characteristics also strongly deviate within supposedly closely related groups. For example, the more basal Phthiracaroidea and Crotonioidea possess strongly diverging claw traits and the higher oribatid mites, also called Brachypylina, also show strong variation with no apparent conformity between supposedly related taxa. Overall, it seems that claw evolution did not primarily follow a phylogenetic pattern. Phylogenetic relationships might still influence specific terrestrial groups, in which attachment and potentially other claw functions are not essential for survival. Nonetheless, to discuss the impact of phylogeny on claws with certainty, future studies must conduct some form of phylogenetic independent contrasts analysis^56^.

In conclusion, our study highlights the complex interplay between environmental, ecological, and functional factors in determining the claw morphology of oribatid mites. While certain observations are consistent with previous studies on arthropods and vertebrates, the distinct ecological contexts and challenges faced by mites necessitate unique morphological adaptations. Further investigations incorporating behavioral, functional, and biomechanical analyses are essential for a more nuanced understanding of the specific benefits and mechanisms underlying these adaptations. Our exploration into the realm of convergent evolution, highlighted by examples such as the adhesive capabilities of geckos and anoles and the sophisticated aquatic feeding strategies of certain mammals, showcases the diverse strategies evolved by various species to overcome environmental challenges^57^. This underscores the profound influence of ecological niches on the evolution of functional anatomical features across a broad spectrum of life, from amphibians to mammals. Such examples of nature's ingenuity in adaptation offer invaluable insights and inspiration for the fields of engineering and material science, driving the development of innovative materials and technologies. The study of biological attachment mechanisms and their ecological and functional contexts remains a fertile ground for future research, promising to further bridge the gap between biology and technology^16^.

### Supplementary Information

Supplementary Information.

## Data Availability

The dataset used and analyzed during the current study is available at 10.5281/zenodo.10837732.
