# Supplementary material for: Multiple factors influence claw characteristics in oribatid mites (Acari)
Source: Sci Rep. 2024 Apr 2;14:7687. doi: 10.1038/s41598-024-58214-4 (PMC10985006; doi:10.1038/s41598-024-58214-4)
Supplement: Supplementary file 1 — Supplementary Information. [file 41598_2024_58214_MOESM1_ESM.docx]

**Supplementary material**

**Multiple factors influence claw characteristics in oribatid mites (Acari)**

Michaela Kerschbaumer^1^* and Tobias Pfingstl ^1^


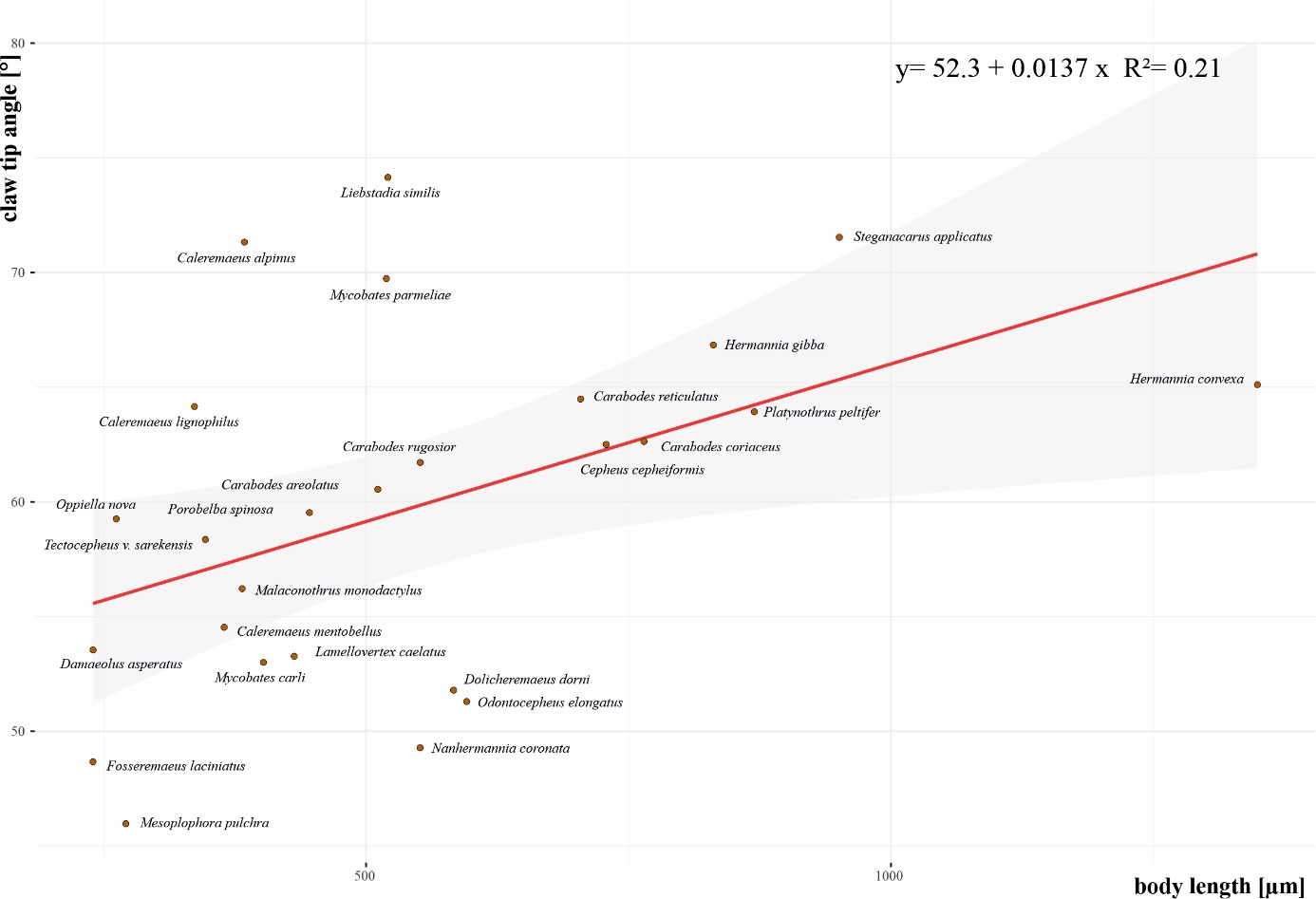


**Figure S1.** Correlation between claw tip angle (in degrees) and body length (micrometers) in terrestrial oribatid mite species. The red line indicates the linear regression model *y= 52.3 + 0.0137 x* with R² value of 0.21.
